# Efficacy and safety of Banxia Xiexin Decoction as a complementary treatment for gastric cancer: A protocol for systematic review and meta-analysis

**DOI:** 10.1097/MD.0000000000025747

**Published:** 2021-04-30

**Authors:** Meiqing Zhang, Wei Huang, Dongkai Yuan

**Affiliations:** Bao’an Authentic Traditional Chinese Medicine Therapy Hospital, Shenzhen, Guangdong Province, PR China.

**Keywords:** Banxia Xiexin Decoction, gastric cancer, meta-analysis, protocol, systematic review

## Abstract

**Background:**

Gastric cancer (GC) is one of the most common malignant tumors in the human digestive system, which seriously affects people's quality of life. As an effective treatment for GC, traditional Chinese medicine can effectively alleviate patients’ clinical symptoms, improve the quality of life, and delay the life cycle. A large number of clinical studies have shown that Banxia Xiexin Decoction has shown a good effect in the treatment of GC. It has achieved good curative effect whether it is used alone or combined with radiotherapy and chemotherapy, which may play a more significant role in the treatment of GC. However, there is still no evidence of evidence-based medicine. Therefore, this study aims to systematically evaluate the efficacy and safety of Banxia Xiexin Decoction as a complementary treatment for GC.

**Methods:**

Two researchers will search the following databases: PubMed, Web of Science, MEDLINE, the Cochrane Library, Embase, China National Knowledge Infrastructure, the Chongqing VIP Chinese Science and Technology Periodical Database, Wanfang Database, and China Biomedical Database. In addition, the Chinese Clinical Trial Register, Chinese Clinical Trial Register, conference papers, and other relevant literature will be searched manually. The retrieval time of these databases is from the establishment of the database to March 2021. The main outcome indicators of this study are the effective rate of treatment and the traditional Chinese medicine syndrome score. According to the inclusion and exclusion criteria of the literature, the data were screened and extracted. The literature quality was evaluated by the bias risk assessment tool of randomized controlled trials recommended by Cochrane Handbook, and meta-analysis was conducted RevMan 5.3 software. The Grading of Recommendations Assessment, Development, and Evaluation will be used to evaluate the quality of evidence.

**Results:**

This study will comprehensively review the existing evidence of Banxia Xiexin Decoction as a complementary in the treatment of GC.

**Conclusion:**

The conclusion of this study will provide a basis for judging whether Banxia Xiexin Decoction is an effective and safe intervention for GC patients.

**Unique INPLASY number:**

INPLASY202140060.

## 1 Introduction

Gastric cancer (GC) refers to malignant tumors (MTs) derived from gastric mucosal epithelial cells, among which gastric adenocarcinoma is the most common, accounting for the leading cause of death of MTs in the world.^[1,2]^ According to the latest cancer statistics, there are more than 1,000,000 new cases and 783,000 deaths of GC in the world in 2018, which is the fifth-largest incidence and the third-largest mortality of MTs.^[3]^ According to Chinese data, GC's incidence and death in China account for 42.6% and 45.0% of GC's global incidence and death, respectively. About 498,000 people die of GC every year, and their incidence ranks third and fourth respectively in male MTs and female MTs, which poses a significant threat to Chinese residents’ life and health.^[4,5]^ As one of the most common MTs in the human digestive system, GC is closely related to infection, diet, environment, and heredity.^[6,7]^ Because the onset of early GC is hidden, there are no clinical symptoms, and the popularity of gastroscopy screening is not high. About 90% of new cases have symptoms and often entered the advanced stage when undergoing clinical screening, which seriously affects patients’ quality of life.^[8]^ As an malignant tumor, it often metastasizes through many ways, such as lymph node metastasis and blood channel metastasis.^[9]^ At present, GC's main treatment methods include surgical resection, chemotherapy, molecular targeted therapy, and immunotherapy, but the prognosis of advanced GC is still not satisfactory due to factors such as easy recurrence, metastasis, and drug resistance.^[10–12]^ Therefore, it is essential for early treatment and prognosis of GC patients to explore the means with adjuvant treatment value actively.

Traditional Chinese medicine (TCM) guides the prevention, diagnosis, and treatment of diseases with holistic concepts and treatment based on syndrome differentiation. As one of the important means to prevent and treat digestive system diseases, TCM has the characteristics of multiple targets and two-way regulation, which plays an important role in the treatment of GC with western medicine, including regulating the immune function of the body, improving the tolerance of surgery and radiotherapy and chemotherapy, improving the preoperative physique, accelerating the postoperative functional recovery, alleviating the toxic and side effects of radiotherapy and chemotherapy, improving the clinical symptoms of patients, preventing and treating tumor recurrence and metastasis, improving the quality of life and delaying the life cycle.^[13–16]^ Banxia Xiexin Decoction comes from the classic book Treatise on Febrile Diseases by Zhang Zhongjing, a famous medical scientist in the Eastern Han Dynasty. The whole prescription is composed of 7 drugs: Ban Xia (Pinellia ternata), Huang Qin (Scutellaria baicalensis georgi), Gan Jiang (Dried ginger), Ren Shen (Ginseng), Huang Lian (Goptis chinensis), Da Zao (Jujube), and Gan Cao (Licorice). It is widely used in the clinical treatment of various gastrointestinal diseases and has certain therapeutic effects on GC.^[17–21]^ Modern studies have also confirmed that Banxia Xiexin Decoction can not only inhibit the proliferation, invasion, and metastasis of GC cells by regulating the cell cycle of GC and promoting the apoptosis of GC cells but also treat precancerous lesions of the stomach and relieve the gastrointestinal reaction of chemotherapy.^[22–26]^

Although many studies have shown that Banxia Xiexin Decoction, whether used alone or combined with radiotherapy and chemotherapy, has shown good effects in the treatment of GC, there is still a lack of large sample, multicenter, randomized, controlled, and double-blind clinical evidence. Therefore, this study uses meta-analysis to evaluate the effectiveness and safety of Banxia Xiexin Decoction adjuvant therapy for GC in order to provide an evidence-based reference for clinical medication.

## 2 Methods

### 2.1 Protocol and registration

The protocol of the systematic review has been registered on the INPLASY website. Registration: INPLASY202140060. https://inplasy.com/inplasy-2021-4-0060/. This study has been drafted under the guidance of the Preferred Reporting Items for Systematic Review and Meta-Analysis Protocols (PRISMA-P).^[27]^

### 2.2 Literature search

Two independent researchers searched the following English and Chinese databases: PubMed, Web of Science, MEDLINE, the Cochrane Library, Embase, China National Knowledge Infrastructure, the Chongqing VIP Chinese Science and Technology Periodical Database, Wanfang Database, and China Biomedical Database. The retrieval time of these databases is from the establishment of the database to March 2021. We will also manually search the Chinese Clinical Trial Register, Chinese Clinical Trial Register, conference papers, and other relevant literature. The retrieval strategy in PubMed is shown in Table 1, and these retrieval strategies are the same in Chinese databases.

**Table 1:** PubMed search strategy.

Number	Search terms
#1	Stomach Neoplasms[MeSH]
#2	Neoplasm, Stomach[title/abstract]
#3	Stomach Neoplasm[title/abstract]
#4	Neoplasms, Stomach[title/abstract]
#5	Gastric Neoplasms[title/abstract]
#6	Gastric Neoplasm[title/abstract]
#7	Neoplasm, Gastric[title/abstract]
#8	Neoplasms, Gastric[title/abstract]
#9	Cancer of Stomach[title/abstract]
#10	Stomach Cancers[title/abstract]
#11	Gastric Cancer[title/abstract]
#12	Cancer, Gastric[title/abstract]
#13	Cancers, Gastric[title/abstract]
#14	Gastric Cancers[title/abstract]
#15	Stomach Cancer[title/abstract]
#16	Cancer, Stomach[title/abstract]
#17	Cancers, Stomach[title/abstract]
#18	Cancer of the Stomach[title/abstract]
#19	Gastric Cancer, Familial Diffuse[title/abstract]
#20	OR #1–#19
#21	Banxia Xiexin Decoction[title/abstract]
#22	randomized controlled trial[Publication Type]
#23	randomized[Title/Abstract]
#24	placebo[Title/Abstract]
#25	OR #22–#24
#33	#20 AND #21 AND #25

### 2.3 Inclusion criteria

(1) Type of studies: We will collect all randomized controlled trials (RCTs) of Banxia Xiexin Decoction as a complementary treatment for gastric cancer. Blind method, language is not limited.
(2) Type of participants: Patients with advanced GC diagnosed by histopathology or clinical diagnosis will be included. Age, sex, course of the disease, source of case, region, and race are not limited.
(3) Types of interventions: The treatment group was treated with Banxia Xiexin Decoction or Banxia Xiexin Decoction combined with routine treatment.
(4) Type of comparators: The control group was given routine treatment, western medicine, or chemotherapy.
(5) Types of outcome measures: This study's main outcome indicators are the effective rate of treatment (adopt the WHO general objective curative effect index of solid tumor) and the TCM syndrome score. Secondary indicators were improved quality of life score, the incidence of adverse reactions (such as nausea, vomiting, diarrhea, bone marrow suppression, leukopenia), recurrence rate, and 2-year survival rate.

### 2.4 Exclusion criteria

(1) Other TCMs were used in the control group;
(2) review, summarization, and repeated publications;
(3) research on incomplete data reporting, duplicate data, or inability to extract data;
(4) animal experimental literature;
(5) documents that do not meet the outcome indicators;
(6) complicated with other severe organs and systemic diseases;
(7) no randomized controlled trial.

### 2.5 Studies selection

**Figure 1:**
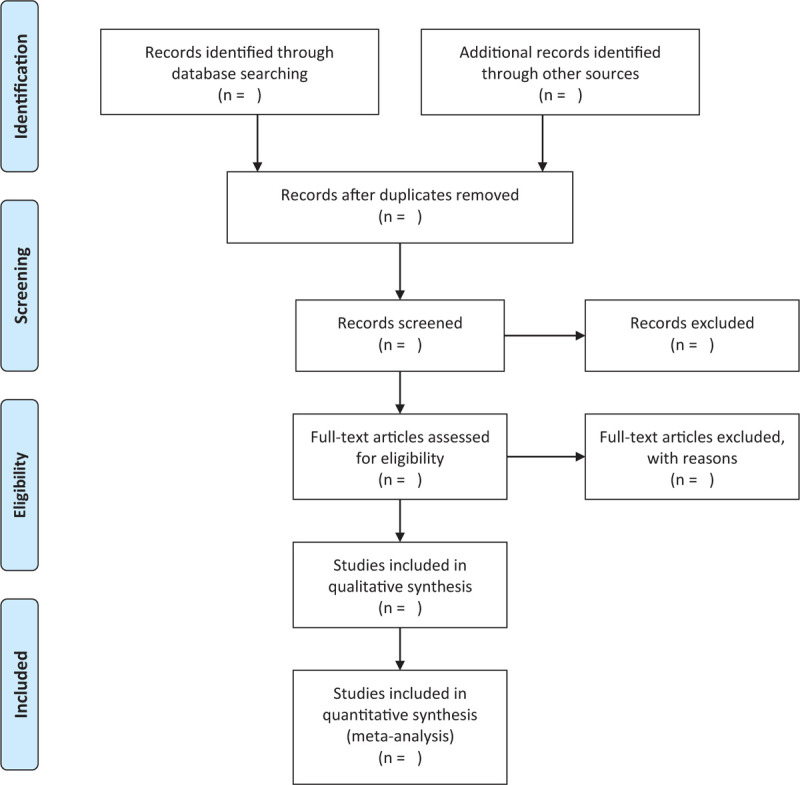
Flow diagram of literature retrieval.

Two researchers independently used EndNote X7 literature management software to screened literature, extracted data, and crosschecked. If there are differences, they will be resolved through discussion or negotiation with a third party. When screening documents, first read the title and then read the abstract and the full text to determine whether or not to include the irrelevant documents. The research selection process is shown in Figure 1 according to the PRISMA flowchart.

### 2.6 Data extraction and management

Two researchers independently extracted and included the literature data. Information will include the author's name, publication year, sample size, number of men and women, age of patients, pathological or clinical stage of gastric cancer, treatment plan, course of treatment, and outcome indicators.

### 2.7 Quality assessment of the studies

Two researchers independently evaluated the bias risk included in the study and crosschecked the results. The randomized controlled trial bias risk assessment recommended by Cochrane Handbook^[28]^ is adopted for the bias risk assessment. The evaluation content mainly includes the following 7 aspects: random sequence generation, allocation hidden, implement of the blind method for patients and testers, implement the blind method for outcome assessors. The resulting data is incomplete, selective reporting, other biases. For each entry, “Yes” stands for “low bias risk,” “No” stands for “high bias risk,” and “unclear” stands for “unclear bias risk.”

### 2.8 Measures of treatment effect

In this study, the risk ratio is used as the measurement result for the counting data. For the measurement data, the mean difference is selected when the measurement results are the same measurement unit. The standardized mean difference is selected as the effect analysis statistic when different measurement units. Each effect quantity provides its effect value and its 95% confidence interval.

### 2.9 Dealing with missing data

If the article's data is missing or insufficient, we will first contact the correspondent author or the first author by email to get complete data. If the author loses the relevant data or cannot contact the author and cannot get complete data, a descriptive analysis will be conducted instead of a meta-analysis.

### 2.10 Assessment of heterogeneity

The heterogeneity of the included studies was tested by *Q* test, and *P* and *I*^2^ values were used as test standards. When *P* > .1 and *I*^2^ ≤ 50%, it was considered that there was no statistical heterogeneity among the studies, and the fixed effect model was used for meta-analysis. On the contrary, when the heterogeneity test result is *P* ≤ .1 and *I*^2^ > 50%, the random effect model is used for meta-analysis.

### 2.11 Data synthesis

Statistical analysis was carried out by RevMan 5.3 software. According to the heterogeneity included in the study, the correlation effect model was selected. If there is apparent clinical heterogeneity, sensitivity analysis or subgroup analysis can be used, or only descriptive analysis can be performed. Draw funnel charts for outcome indicators with more than 10 studies, and analyze publication bias.

### 2.12 Sensitivity analysis

To judge the stability of the data synthesis, we will probably use sensitivity analysis for the data.

### 2.13 Subgroup analysis

If there is a heterogeneity source, we will make a subgroup analysis of this reason and then see if there is significant heterogeneity.

### 2.14 Summary of evidence

The Grading of Recommendations Assessment, Development, and Evaluation will be used to evaluate the quality of evidence for each result from the meta-analysis.^[29]^ According to the effect of Banxia Xiexin Decoction as a complementary in gastric cancer treatment, the evaluation results will be divided into high quality, medium quality, low quality, and very low quality.

### 2.15 Ethics and dissemination

Ethical approval is not required for this protocol because there is no patient recruitment and personal information collection. The results of this systematic review will be published in a peer-reviewed journal and relevant conference presentations.

## 3 Discussion

At present, GC, as one of the chronic diseases threatening life and health, has a high degree of malignancy and poor prognosis. With the changes in population structure, geographical environment, and eating habits, the incidence of GC will gradually become younger. Therefore, it is still necessary to explore the treatment of GC. GC can belong to the categories of “Zhengjia” and “Weiwantong” in TCM. TCM plays a vital role in the treatment of MTs and has gradually become a critical consensus in the treatment of MTs. For patients with GC, we can strengthen the body resistance and eliminate pathogenic factors, treat both the symptoms and root causes, and treat them according to their physical condition to improve their clinical symptoms, improve their quality of life, and reduce the side effects of radiotherapy and chemotherapy. In recent years, many clinical RCTs related to Banxia Xiexin Decoction in treating GC have been published. Therefore, we hope to discuss the effectiveness and safety of Banxia Xiexin Decoction as a complementary in the treatment of GC by comprehensively analyzing the research literature of Banxia Xiexin Decoction in treating GC and provide a reference for clinical practice.

## Author contributions

**Conceptualization:** Meiqing Zhang, Wei Huang, Dongkai Yuan.

**Data curation:** Meiqing Zhang, Wei Huang.

**Formal analysis:** Meiqing Zhang, Dongkai Yuan.

**Investigation:** Meiqing Zhang, Wei Huang, Dongkai Yuan.

**Methodology:** Wei Huang, Dongkai Yuan.

**Software:** Wei Huang, Dongkai Yuan.

**Supervision:** Meiqing Zhang, Wei Huang.

**Writing – original draft:** Meiqing Zhang, Wei Huang.

**Writing – review & editing:** Meiqing Zhang, Dongkai Yuan.

## References

[1] Siegel RL Miller KD Jemal A. Cancer statistics. CA Cancer J Clin 2018;68:07–30. 10.3322/caac.21442 29313949

[2] Bray F Ferlay J Soerjomataram I. Global cancer statistics 2018: GLOBOCAN estimates of incidence and mortality worldwide for 36 cancers in 185 countries. CA Cancer J Clin 2018;68:394–424. 30207593 10.3322/caac.21492

[3] Sitarz R Skierucha M Mielko J. Gastric cancer: epidemiology, prevention, classification, and treatment. Cancer Manag Res 2018;10:239–48. 29445300 10.2147/CMAR.S149619 PMC5808709

[4] Chen W Zheng R Baade PD. Cancer statistics in China, 2015. CA Cancer J Clin 2016;66:115–32. 26808342 10.3322/caac.21338

[5] Ferlay J Soerjomataram I Dikshit R. Cancer incidence and mortality worldwide: sources, methods and major patterns in GLOBOCAN 2012. Int J Cancer 2015;136:E359–86. 25220842 10.1002/ijc.29210

[6] Zuo TT Zheng RS Zeng HM. Epidemiology of stomach cancer in China. Chin J Clin Oncol 2017;44:52–8.

[7] Hamashima C. Current issues and future perspectives of gastric cancer screening. World J Gastroenterol 2014;20:13767–74. 25320514 10.3748/wjg.v20.i38.13767 PMC4194560

[8] Du YQ Cai QC Liao Z. Expert consensus on screening process of early gastric cancer in China. Chin J Dig Endoscopy 2018;35:77–83.

[9] Chu ZW Liu YQ. Effect of traditional Chinese medicine in treatment of lymphatic metastasis of gastric cancer. Chin J Exp Tradit Med Form 2020;26:225–31.

[10] Lazăr DC Tăban S Cornianu M. New advances in targeted gastric cancer treatment. World J Gastroenterol 2016;22:6776–99. 27570417 10.3748/wjg.v22.i30.6776 PMC4974579

[11] Yu J Huang C Sun Y. Effect of laparoscopic vs open distal gastrectomy on 3-year disease-free survival in patients with locally advanced gastric cancer: the CLASS-01 randomized clinical trial. JAMA 2019;321:1983–92. 31135850 10.1001/jama.2019.5359 PMC6547120

[12] Cats A Jansen EPM Van Grieken NCT. Chemotherapy versus chemoradiotherapy after surgery and preoperative chemotherapy for resectable gastric cancer (CRITICS): an international, open-label, randomised phase 3 trial. Lancet Oncol 2018;19:616–28. 29650363 10.1016/S1470-2045(18)30132-3

[13] Cui GN Liu XP Dong JG. Research progress of Banxia Xiexin Decoction in the treatment of gastric cancer. J Basic Chin Med 2020;26:1905–8.

[14] Xie CS Wang YK Lou JJ. Progress of experimental research on TCM treatment of gastric carcinoma. Chin Arch Tradit Chin Med 2015;33:2743–5.

[15] Feng Y Wu CY Li J. Promising role and probable molecular mechanisms of TCM in gastric cancer treatment. Liaoning J Tradit Chin Med 2017;44:200–3.

[16] Chen H Lv SQ. General situation of research on TCM treatment of gastric cancer. Xinjiang J Tradit Chin Med 2019;37:76–7.

[17] Li J Jia ZZ. Research progress on clinical application of Banxia Xiexin Decoction. Hunan J Tradit Chin Med 2018;34:185–8.

[18] Dai HJ. Revised pinellia combination treat digestive tract reaction from chemical therapy after operation of gastric cancer. Guangming J Chin Med 2020;35:2376–8.

[19] Gu WJ. Flavored Banxia Xiexin Decoction combined with chemotherapy on patients life quality after operation. Acta Chin Med 2015;30:626–8.

[20] Feng LL Zhang AP Dong YP. Application of Banxia Xiexin Decoction in prevention and treatment of gastric cancer. Chin J Exp Tradit Med Form 2012;18:258–9.

[21] Liu NN Tan Z Sun MY. Research progress of Banxia Xiexin Decoction on gastric cancer. Chin J Tradit Chin Med Pharm 2020;35:6254–7.

[22] Zhang W Liu XP Ming HX. Effects of drug serum of Banxia Xiexin Decoction on peritoneal mesothelial HMrSV5 cell apoptosis induced by gastric cancer microenvironment. Chin J Tradit Chin Med Pharm 2016;31:3735–8.

[23] Yang BL Liu XP Cui GN. Effect of serum containing Banxia Xiexin Tang on growth and proliferation of bone mesenchymal stem cells in gastric carcinoma microenvironment. Chin J Tradit Chin Med Pharm 2016;22:97–102.

[24] Cui GN Liu XP Li PQ. Influence of drug serum in pinellisa decoction for purging stomach-fire on peritoneal mesothelial cell oxidative stress by gastric cancer microenvironment. Lishizhen Med Mater Med Res 2017;28:12–4.

[25] Kim HR Lee GS Kim MS. Effects of Banxia Xiexin Decoction on cisplatin-induced apoptosis of human A549 lung cancer cells. Chin J Integr Med 2018;24:436–41. 29247342 10.1007/s11655-017-2922-x

[26] Yin KK Cao R Tang B. Effect of Banxia Xiexin Decoction on gastric microorganisms and enzymes in mice with gastritis infected by helicobacter pylori. World Chin J Dig 2014;22:3067–71.

[27] Shamseer L Moher D Clarke M. Preferred reporting items for systematic review and meta-analysis protocols (PRISMA-P) 2015: elaboration and explanation. BMJ 2015;350:01–25. 10.1136/bmj.g7647 25555855

[28] Higgins JP Altman DG Gotzsche PC. The Cochrane Collaboration's tool for assessing risk of bias in randomised trials. BMJ 2011;343:01–9. 10.1136/bmj.d5928 PMC3196245 22008217

[29] Deng T Wang Y Huang D. Methods for formulating clinical practice guidelines: GRADE method theory. Chin J Evid Based Cardiovasc Med 2018;10:1441–5.

